# CircDONSON regulates the proliferation, invasion and migration of non-small cell lung cancer cells through the MAPK signaling pathway

**DOI:** 10.1016/j.gendis.2024.101217

**Published:** 2024-01-23

**Authors:** Shichao Zhang, Zhenguo Zeng, Feng Qiu, Xiaolei Li, Xinping Xu

**Affiliations:** a Jiangxi Institute of Respiratory Disease, The Department of Respiratory and Critical Care Medicine, The First Affiliated Hospital, Jiangxi Medical College, Nanchang University, Nanchang, Jiangxi 330006, China; b Department of Intensive Care Unit, The First Affiliated Hospital, Jiangxi Medical College, Nanchang University, Nanchang, Jiangxi 330006, China; c Gaoxin Hospital, The First Affiliated Hospital, Jiangxi Medical College, Nanchang University, Nanchang, Jiangxi 330006, China; d China-Japan Friendship Jiangxi Hospital, National Regional Center for Respiratory Medicine, Nanchang, Jiangxi 330200, China

Lung cancer is an exceedingly prevalent form of cancer, frequently diagnosed, and holds the unfortunate distinction of being the primary cause of cancer-related fatalities on a global scale.^1^ Non-small cell lung cancer (NSCLC) accounts for 85 % of all lung cancer cases and has a low 5-year survival rate.^2^ Previous studies have revealed that CircDONSON plays a pivotal role in promoting the growth and invasion of gastric cancer by activating the NURF complex-dependent transcription factor SOX4.^3^ However, the function significance of CircDONSON in lung cancer remains unexplored. Mechanistically, we demonstrate that CircDONSON interacts with HNRNPC and subsequently inhibits the downstream MAPK signaling pathway. To summarize, our study demonstrates that CircDONSON acts as a tumor suppressor in lung cancer and exhibits potential as both a prognostic marker and therapeutic target for NSCLC.

CircDONSON (circbase ID: hsa_circ_0004339) is located on chromosome 21q22.11, spanning a length of 948 nucleotides. It is generated through the back-splicing process involving exon 3 to exon 8 of DONSON pre-mRNAs (Fig. 1A). To determine whether CircDONSON is related to non-small cell lung cancer, we initially utilized quantitative reverse transcription PCR to analyze the expression disparity of CircDONSON in non-small cell lung cancer tissues versus adjacent normal tissues (Fig. 1C). Subsequently, we assessed the expression of CircDONSON in various non-small cell lung cancer cell lines (Fig. 1B). The findings revealed that the expression of CircDONSON in lung cancer cell lines was notably lower than that of Beas-2b cells (Fig. 1B). Certainly, we verified circDONSON's circular RNA signature using Sanger sequencing, PCR analysis, and RNase R exonuclease digestion assay (Fig. S1). Based on our results, it can be inferred that CircDONSON is down-regulated in NSCLC and potentially plays a role in regulating multiple biological functions related to NSCLC.

**Figure 1:**
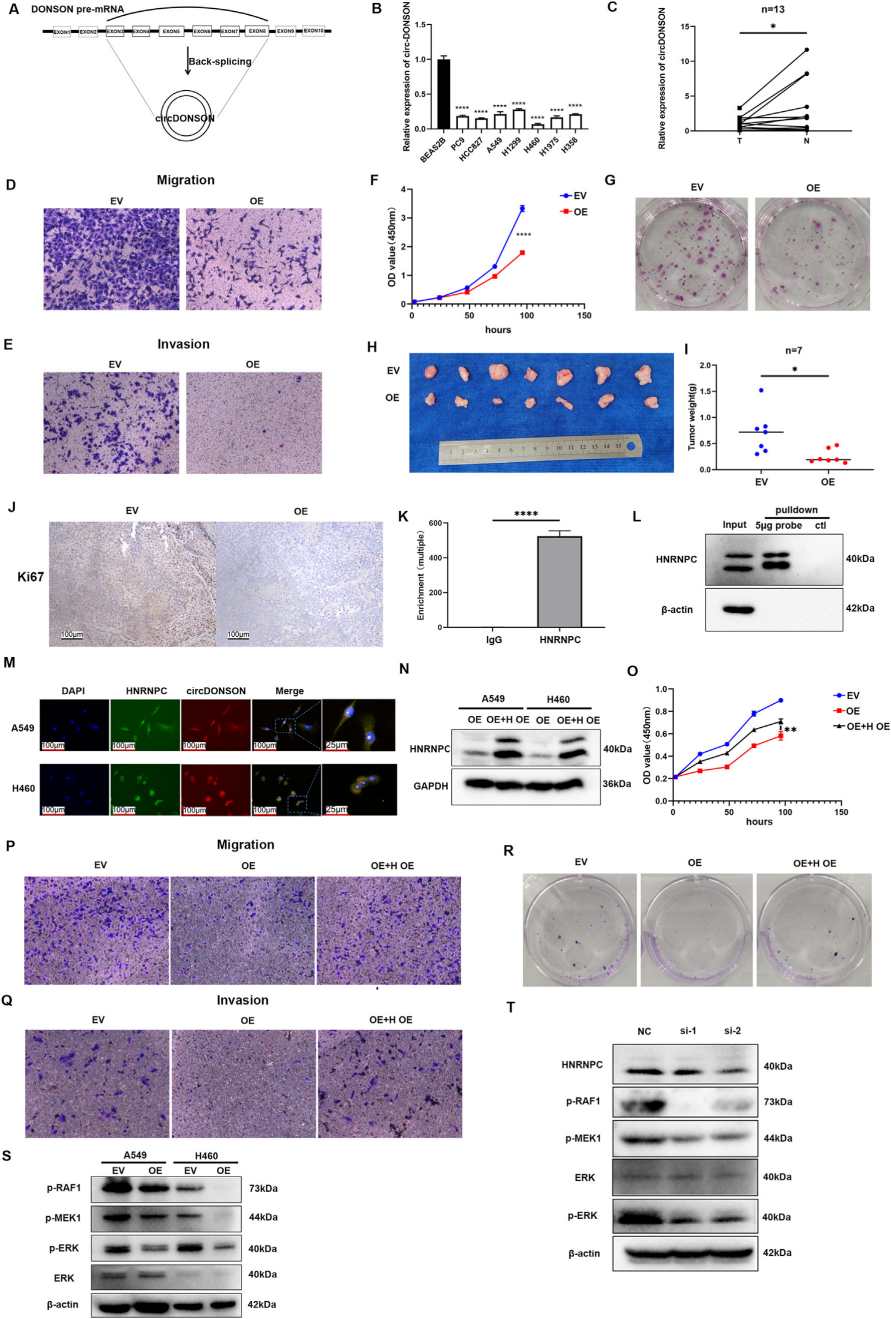
CircDONSON regulates the proliferation, invasion, and migration of non-small cell lung cancer (NSCLC) cells through the MAPK signaling pathway. **(A)** Structural diagram of CircDONSON. **(B)** The level of CircDONSON was decreased in NSCLC cell lines versus Beas-2b cells. **(C)** The level of CircDONSON was decreased in NSCLC tissues versus para-carcinoma tissues. **(D)** The representative images of Transwell assays showing the migration of A549 cells stably transfected with empty vector (EV) and overexpression vector carrying circDONSON. **(E)** Representative images of Matrigel assays showing the invasion of A549 cells stably transfected with empty vector (EV) and circDONSON. **(F)** The proliferation of A549 cells with overexpression of CircDONSON was analyzed with CCK-8. **(G)** Colony formation assays were applied to detect the proliferation of A549 cells with overexpression of CircDONSON. **(H)** Photo of tumors separated from nude mice transplanted with A549 carrying empty vector (EV) or CircDONSON (OE). **(I)** The tumor weight was measured 19 days after injection. **(J)** Immunohistochemistry analysis of Ki67 expression in tumor tissues of each group. **(K, L)** RNA immunoprecipitation and RNA pulldown experiments demonstrated the interaction between CircDONSON and HNRNPC. **(M)** Immunofluorescence showed that CircDONSON and HNRNPC were co-localized in the nucleus. **(N)** HNRNPC protein was overexpressed in stable overexpressing CircDONSON cell lines. **(O, R)** CCK-8 assay and clonal formation assay were used to analyze the proliferation ability of the three groups of cells. **(P, Q)** Transwell assay showed that overexpression of HNRNPC promoted migration and invasion of the two groups of cells. **(S, T)** Overexpression of CircDONSON and knockdown of HNRNPC inhibited the MAPK signaling pathway.

In order to study the biological function of CircDONSON in NSCLC, we constructed a CircDONSON overexpression vector and transfected it into NSCLC cells. Our data show that overexpressed CircDONSON could not only inhibit cell migration and invasion significantly (Fig. 1D, E; Fig. S2D, E) but also inhibit cell proliferation and colony formation ability (Fig. 1F, G; Figs. S2A–C). These results indicate that overexpression of CircDONSON suppresses NSCLC cell progression *in vitro*.

To further explore the function of CircDONSON, we performed tumor formation experiments in Balb-c nude mice. The results showed that the tumor volume and weight from the CircDONSON overexpression group were significantly lower than those from the empty plasmid control group (Fig. 1H, I; Fig. S3). Ki67 staining further proved the significantly lower proliferation ability in CircDONSON overexpression group (Fig. 1J). Taken together, these suggest that CircDONSON can inhibit the progression of NSCLC *in vivo*.

Next, we investigated the potential mechanism by which circDONSON inhibited NSCLC progression. We utilized the http://circatlas.biols.ac.cn/database to predict CircDONSON potential binding proteins. Additionally, proteins harvested by circDONSON pulldown were identified by mass spectrometry or liquid chromatography-mass spectrometry, and five specific proteins were picked through intersection analysis (Fig. S4A). To further explore the interaction between CircDONSON and specific proteins, we conducted RNA pulldown and RNA immunoprecipitation experiments to confirm the interaction between HNRNPC and CircDONSON (Fig. 1K, L). Immunofluorescence assay further demonstrated colocalization of CircDONSON and HNRNPC in the nucleus (Fig. 1M). Analysis of data from the TCGA database has revealed a high expression of HNRNPC in NSCLC, which is associated with poor prognosis of NSCLC patients (Fig. S5A, B). Our data showed that silencing HNRNPC could impair the growth, migration, and invasion abilities (Figs. S5C–G), as reported previously.^4^

Interestingly, when HNRNPC was overexpressed in CircDONSON overexpressing lung cancer cell lines, a restoration in the proliferation, invasion, and migration was observed (Fig. 1N–R; Fig. S6). In order to explore the specific signaling pathways of circDONSON inhibiting NSCLC, we tested the level of proteins in the MAPK signaling pathway in cell lines with CircDONSON overexpression. The results demonstrated a significant reduction in the expression levels of MAPK signaling pathway-related proteins in the overexpression group (Fig. 1S). Interestingly, we also observed decreased protein levels in the MAPK pathway after HNRNPC knockdown (Fig. 1T).

In conclusion, it is worth emphasizing that the circular RNA CircDONSON exhibits a notable tumor suppressor effect in NSCLC, representing a remarkable biomarker and a potent therapeutic target. The outcomes of functional experiments have furnished compelling evidence, demonstrating that CircDONSON effectively regulates the proliferation, migration, and invasion of NSCLC cells. This regulatory mechanism is accomplished by binding the HNRNPC protein, thereby inhibiting the MAPK signaling pathway (Fig. S7).

## Ethics declaration

All procedures performed in this study involving human samples were in accordance with the ethical standards of the Ethics Committee of the First Affiliated Hospital of Nanchang University (Ethics: (2022) CDYFYYLK (07–005)). Animal protocols were approved by the Ethics Committee of the First Affiliated Hospital of Nanchang University (CDYFY-IACUC202210QR009).

## Author contributions

All authors contributed to the design and implementation of this study. Z.S. and L.X. performed experiments and acquired data; Z.Z., Q.F., and X.X. performed database analyses and supervised the study. Z.S., L.X., and X.X. analyzed the data and wrote the manuscript. All authors reviewed and commented on the final manuscript.

## Conflict of interests

The authors declare no conflict of interests.

## Funding

This work was supported by the National 10.13039/501100004479Natural Science Foundation of Jiangxi Province, China (No. 20212BAB206034), Innovative and Entrepreneurial Youth Talents Project of Jiangxi Province, China (No. JXSQ2018106040), and Nanchang Key Laboratory of Tumor Gene Diagnosis and Innovative Treatment Research (China) (No. 2021—NCZDSY-009).
