# Supplementary material for: CircDONSON regulates the proliferation, invasion and migration of non-small cell lung cancer cells through the MAPK signaling pathway
Source: Genes Dis. 2024 Jan 23;12(1):101217. doi: 10.1016/j.gendis.2024.101217 (PMC11472607; doi:10.1016/j.gendis.2024.101217)
Supplement: Multimedia component 1 [file mmc1.docx]

**Figure legend**

FigureS1. Characterization of circDONSON in NSCLC cell lines. A, The PCR products amplified by qRT-PCR primers were subsequently sequenced. B, The PCR analysis of CircDONSON and GAPDH from cDNA and genomic DNA. RH random hexamers, OdT oligo(dT)18 primers, gDNA genomic DNA. C and D, The RNA levels of circDONSON and DONSON after treatment with RNase R. (Data are presented as mean ± s.d. Differences in (C, D) were compared using the student T-test and analysis of variance. **P*<0.05, *****P*<0.0001.)

FigureS2: Overexpression of circDONSON can inhibit the proliferation, migration and invasion of NSCLC cells. A and B, A549 and H460 cell proliferation with CircDONSON overexpression was assessed using CCK-8. C, Colony formation assays were applied to detect the proliferation of A549 and H460 cells with overexpression of CircDONSON. D, Representative images (left panel) and quantification (right panel) of Transwell assays, respectively, showing the migration of A549 cells and H460 cells stably transfected with empty vector (EV) and overexpression vector carrying circDONSON. E, Representative images (left panel) and quantification (right panel) of Matrigel assays, respectively, showing the invasion of A549 cells and H460 cells stably transfected with empty vector (EV) and circDONSON. (**P*<0.05, ***P*<0.01, ****P*<0.001,*****P*<0.0001.)

FigureS3: Overexpression of circDONSON inhibits the growth of NSCLC tumors in mice. Tumor size were determined every 3 days.

FigureS4: circDONSON is associated with HNRNPC. A, Venn diagram shows the overlap proteins between the result of prediction from circatlas database and result from RIP combined with LC-MS/MS.

FigureS5: HNRNPC is highly expressed in NSCLC and promotes the proliferation, migration and invasion of NSCLC cells. A and B, Analysis from TCGA database showed that HNRNPC is highly expressed in NSCLC and is associated with poor prognosis of NSCLC patients. C, The expression of HNRNPC was knocked down by small interfering RNA transfection. D and E, CCK-8 assay were used to analyze the proliferation of A549 and H460 cells with HNRNPC knockdown. F and G, Transwell assay showed that knockdown of HNRNPC inhibited migration and invasion of A549 and H460 cells. (**P*<0.05, ***P*<0.01, ****P*<0.001,*****P*<0.0001. )

FigureS6: High expression of HNRNPC in NSCLC cells overexpressing circDONSON can restore the proliferation, migration and invasion ability of NSCLC cells. A-C, CCK-8 assay and Clonal formation assay were used to analyze the proliferation ability of the two groups of cells.D-E: Transwell assay showed that overexpression of HNRNPC promoted migration and invasion of the three groups of cells. (**P*<0.05, ***P*<0.01, ****P*<0.001, *****P*<0.0001.)

FigureS7: Working model-CircDONSON binds to HNRNPC to affect the MAPK signaling pathway.
