# Supplementary material for: CircDONSON regulates the proliferation, invasion and migration of non-small cell lung cancer cells through the MAPK signaling pathway
Source: Genes Dis. 2024 Jan 23;12(1):101217. doi: 10.1016/j.gendis.2024.101217 (PMC11472607; doi:10.1016/j.gendis.2024.101217)
Supplement: Multimedia component 2 [file mmc2.pdf]

Figure S1

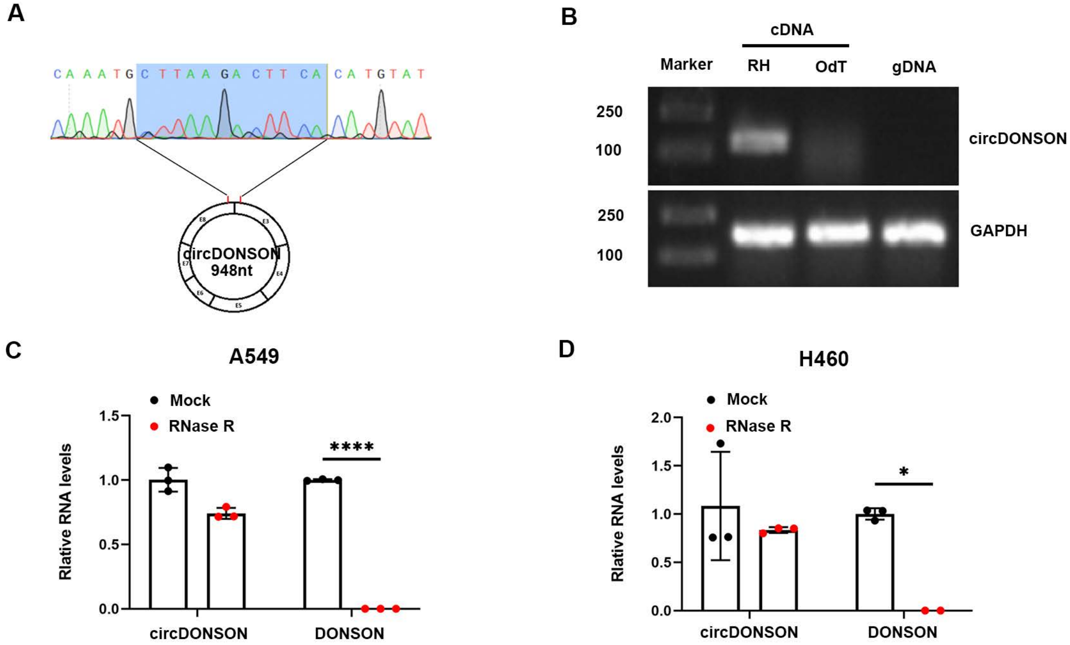
A
C A A A T G C T T A A G A C T T C A C A T G T A T
circDONSON
948nt
E3
E4
E5
E6
E7
E8
B
cDNA
Marker
RH
OdT
gDNA
250
100
circDONSON
250
100
GAPDH
C
A549
Mock
RNase R
****
Rlative RNA levels
1.5
1.0
0.5
0.0
circDONSON
DONSON
D
H460
Mock
RNase R
*
Rlative RNA levels
2.0
1.5
1.0
0.5
0.0
circDONSON
DONSON

Figure S2

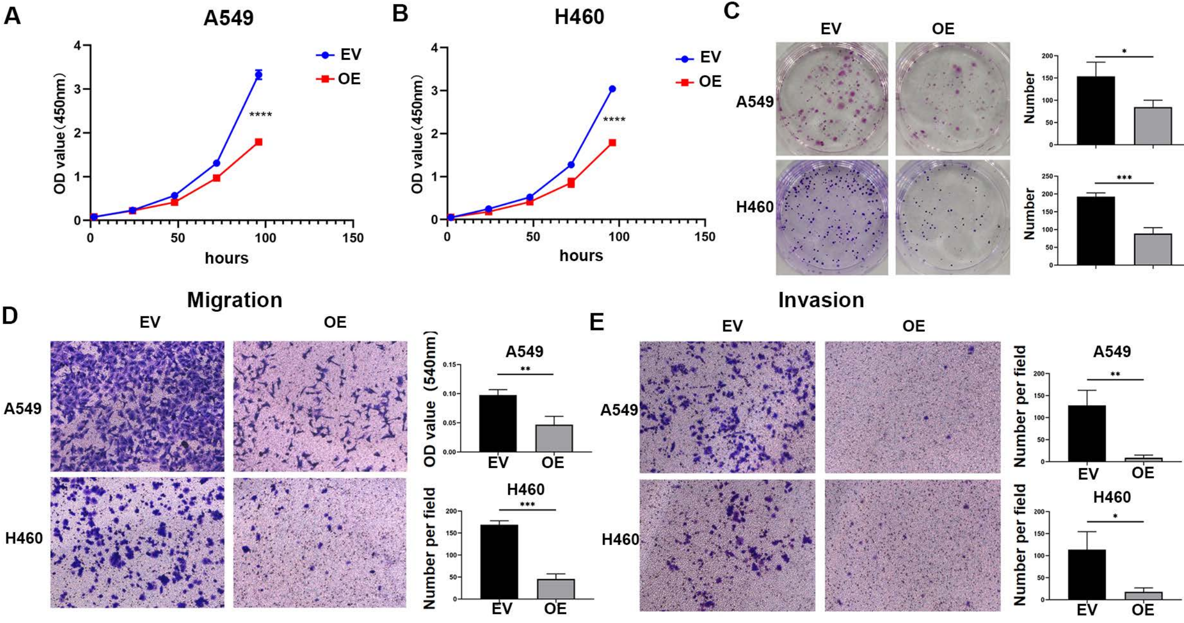

A
A549
OD value（450nm）
hours
EV
OE
****
B
H460
OD value（450nm）
hours
EV
OE
****
C
EV
OE
A549
H460
Number
*
***
Migration
D
EV
OE
A549
H460
OD value（540nm）
Number per field
**
***
Invasion
E
EV
OE
A549
H460
Number per field
**
*

Figure S3

A

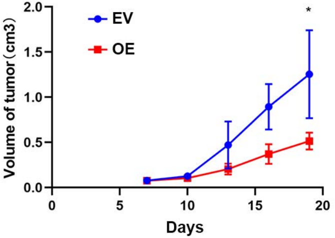

Figure S4

A

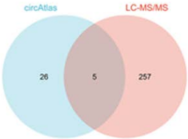

Gene	Intensity
HNRNPC	24150000
U2AF1	23246000
HNRNPU	21640000
PTBP1	18916000
NOP56	0

Figure S5

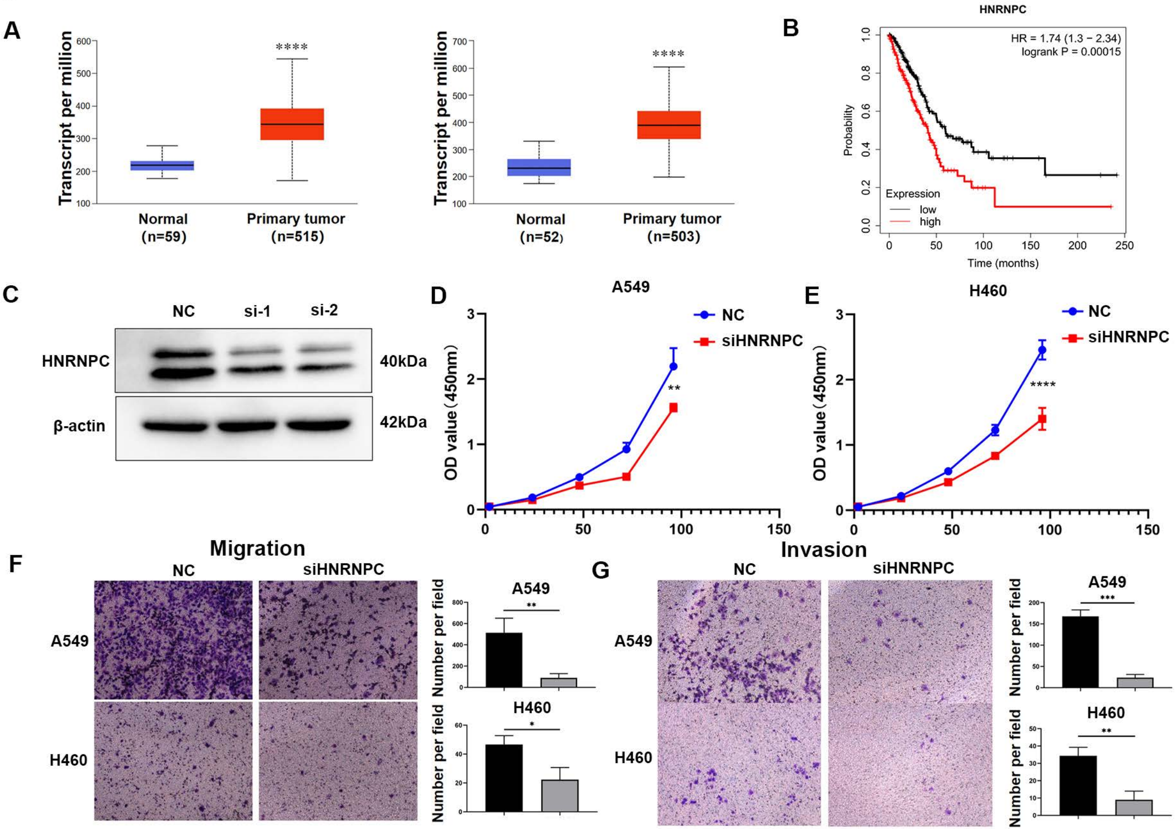

A
Transcript per million
Normal (n=59)
Primary tumor (n=515)
Normal (n=52)
Primary tumor (n=503)
B
HNRNPC
HR = 1.74 (1.3 − 2.34)
logrank P = 0.00015
Probability
Time (months)
Expression
low
high
C
NC
si-1
si-2
HNRNPC
β-actin
40kDa
42kDa
D
A549
E
H460
NC
siHNRNPC
OD value（450nm）
Migration
F
Invasion
G
NC
siHNRNPC
A549
H460
Number per field

Figure S6

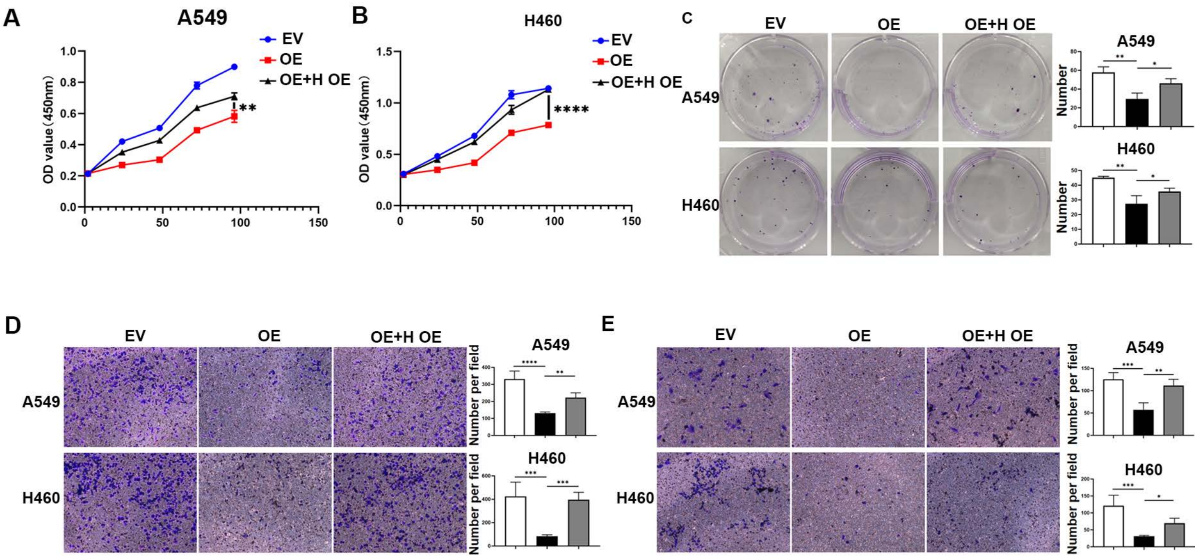
A
A549
EV
OE
OE+H OE
OD value (450nm)
**
B
H460
****
C
EV
OE
OE+H OE
A549
H460
Number
D
E
Number per field

Figure S7

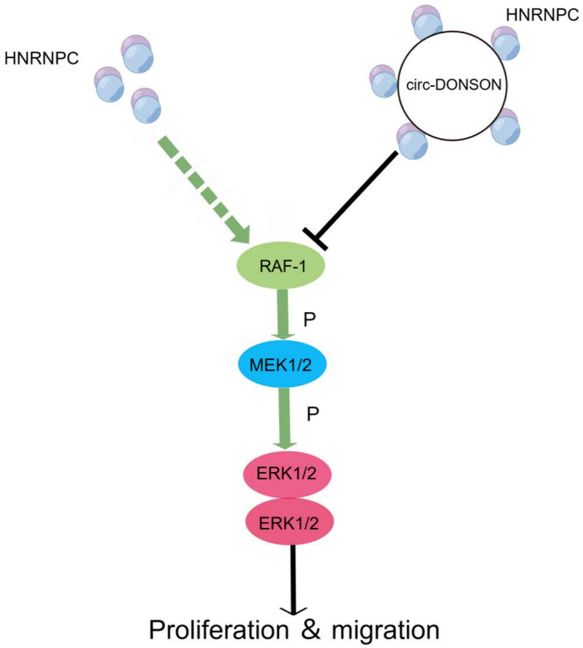
HNRNPC
HNRNPC
circ-DONSON
RAF-1
P
MEK1/2
P
ERK1/2
ERK1/2
Proliferation & migration
