# Supplementary material for: CircDONSON regulates the proliferation, invasion and migration of non-small cell lung cancer cells through the MAPK signaling pathway
Source: Genes Dis. 2024 Jan 23;12(1):101217. doi: 10.1016/j.gendis.2024.101217 (PMC11472607; doi:10.1016/j.gendis.2024.101217)
Supplement: Multimedia component 3 [file mmc3.doc]

**Materials and methods**

**Tissues**

A total of 13 pairs of primary NSCLC tumor tissue (T) and adjacent non-tumor tissue (N) were collected from patients who underwent surgery at the First Affiliated Hospital of Nanchang University between May 2021 and June 2022. All 13 tissues were used for qRT-PCR validation. The samples were stored at -80 °C until use. The collection of all tissue specimens was conducted between May 2021 and June 2022 and was approved by the Ethics Committee of the First Affiliated Hospital of Nanchang University (Ethics: (2022) CDYFYYLK (07-005)).

**Cell culture**

The human NSCLC cell lines BEAS-2B, H460, PC9, HCC827, H358 and H1299 were purchased from ATCC (Procell Life Science&Technology Co., Ltd) and maintained in RPMI-1640 (Gibco), except for A549, which was maintained in DMEM (Gibco). Both media were supplemented with 10% fetal bovine serum (FBS; Life Technologies). The cells were incubated at 37℃ in a humidified atmosphere with 5% CO_2_.

**Quantitative real-time PCR (qRT-PCR)**

Total RNA was isolated using TransZol Up reagent (Transgen Biotech). The quantification of circRNA was performed using Hifair III 1st Strand cDNA Synthesis SuperMix for qPCR (YEASEN Biotech). Prior to calculation using the ΔΔCt method, the circRNA level in the nucleus was normalized using the levels of small nuclear U6.

**Vector construction and cell transfection**

Schematic diagrams of CircDONSON overexpression and empty plasmid construction were presented in Figure S1. Briefly, for the CircDONSON overexpression version, the entire 948bp sequence of hsa_circ_0004339 was amplified using PCR, and the in-fusion clone was connected to PLC5-CIR (Geneseed, Guangzhou, China). The overexpressed plasmids were transfected using the Lentiviral Packaging Kit (YEASEN Biotech) following the manufacturer's instructions.

**CCK-8** **assay**

Cell proliferation was assessed by cell counting kit -8 (YEASEN Biotech). 3000 cells per well were seeded into 96-well plates and treated as experiment requirement. At the end of treatment, 10 μL CCK-8 solution was added into each well (containing 100μL fresh culture media) and incubate for 2-hour at 37℃ with 5% CO_2_.  450nm absorbance was s measured using a spectrophotometer (Thermo scientific). Cell proliferation rate was calculate as following :

**Colony formation assay**

A549 and H460 cells were planted into the 6-well plates(5×10^2^ cells/well) and incubated for 10 days at 37 °C. Then colonies were fixed with 6% glutaraldehyde(v/v) and stained with 0.1%(w/v) crystal violet. The colony numbers were counted finally.

**Transwell assay**

The migration and invasion tests share similar methods. The transwell chamber (Corning, USA) was positioned in a 24-well plate. The dissimilarity between the two methods lies in the membrane type: the invasion test utilized a matrigel gel-coated transwell chamber (YEASEN Biotech), while a regular membrane was used for the migration test. For the migration experiment, cells were suspended in 200μL of 0.1% serum medium, inoculated in the upper cavity, and 10% fetal bovine serum medium was added to the lower cavity. Following incubation for 16 hours (A549) and 24 hours (H460), the cells were fixed onto the membrane using a fixative solution. Subsequently, the cells were stained with 0.5% crystal lavender (Solarbio), gently removing the cells from the upper surface of the transwell chamber with a cotton swab. Finally, four microscope fields were randomly selected for cell counting.

**RNA immunoprecipitation.**

RIP experiments were performed with a Magna RIP RNA-Binding Protein Immunoprecipitation Kit (Millipore, Billerica, MA, USA) in accordance to the manufacturer’s instructions. The co-precipitated RNA was then detected using qRT-PCR.

**RNA pulldown assay**

A total of 1 × 10^7^ lung cancer cells were harvested and cleaved. Subsequently, the CircDONSON probe was incubated with magnetic beads (BersinBio, Guangzhou, China) at 25°C for 2 h to generate microspheres coated with the probe. The cell lysates were then incubated overnight at 4°C either with CircDONSON probe or negative control probe. After washing with washing buffer, protein loading buffer was added to the magnetic beads, and the mixture was heated at 95℃for 10 minutes. The magnetic beads were discarded, and the protein solution was used for Western blotting.

**Western blotting analysis**

Total protein was extracted from cells using radioimmunoprecipitation assay RIPA lysis buffer (Applygen). A 1:100 dilution of Protease inhibitor Cocktail and a 1:100 dilution of Halt Phosphatase Inhibitor Cocktail (Thermo Scientific) and PMSF (Shanghai Epizyme Biomedical Technology) were added according to the manufacturer’s instructions. The protein concentration was determined using BCA Protein Assay Kit (GLPBIO). Equal amounts of extracts were loaded onto SDSPAGE gel for electrophoresis. After separation on the gel, the protein was transferred to the polyvinylidene fluoride (PVDF) membrane. The membrane was then blocked with 5% milk in TBS-T buffer solution for 1h, and then incubated overnight at 4℃ with antibodies against HNRNPC (proteintech), p-ERK (abcam), ERK (abcam), RAF-1(abcam), MEK-1(abcam)and β-Tublin (proteintech).

**Animal assay**

The animal experiments conducted in this study were approved by the Ethics Committee of the First Affiliated Hospital of Nanchang University. To establish a stable cell line that overexpresses circDONSON, the corresponding lentivirus was transduced into H460 and A549 cells. Following transduction, the cells were screened using purinamycin. As controls, cells transfected with empty plasmids were utilized. For the tumor formation experiment of nude mice, 5×10^6^ modified H460 cells were subcutaneously inoculated into the left and right back of 10 male Balb/c nude mice. The tumor volume was measured every 3 days and calculated as π/6× length × width ^2^. After 19 days, the mice were sacrificed, and tumor tissues were weighed and collected for RT-qPCR and immunostaining analysis.

**Statistical analysis**

Statistical analyses were performed using GraphPad Prism 8.0. The results are presented as the mean±SEM. The statistical significance between two groups was assessed with Student’s t test (unpaired, two-tailed), whereas the statistical significance among multiple groups was analyzed using ANOVA followed by Bonferroni’s multiple comparison test. A *p*-value less than 0.05 was considered statistically significant. All biochemical experiments and representative images were performed in a minimum of three independent experiments.
