# Supplementary material for: CircDONSON regulates the proliferation, invasion and migration of non-small cell lung cancer cells through the MAPK signaling pathway
Source: Genes Dis. 2024 Jan 23;12(1):101217. doi: 10.1016/j.gendis.2024.101217 (PMC11472607; doi:10.1016/j.gendis.2024.101217)
Supplement: Multimedia component 4 [file mmc4.docx]

Table1. Sequences of Primers and siRNAs used in the text

| Gene | Sequence |
| --- | --- |
| circDONSON | F（5'-3'）CACAATGCAAATGCTTAAGACT |
|  | R（5'-3'）GTTGAGAAGAGGTGAAAAGGAG |
| DONSON | F（5'-3'）CCTGGTGGCGGGGCTGCCTCTT |
|  | R（5'-3'）CTTCCCATAGCAAATCATTTTCTTGA |
| GAPDH | F（5'-3'）CCCACATGGCCTCCAAGGAGTA |
|  | R（5'-3'）GTGTACATGGCAACTGTGAGGAGG |
| U6 | F（5'-3'）CTCGCTTCGGCAGCACA |
|  | R（5'-3'）AACGCTTCACGAATTTGCGT |
| siHNRNPC 1 | 5'-3' GCCUUCGUUCAGUAUGUUAAU |
| siHNRNPC 2 | 5'-3' CAGAUCGUUUCACACCUGCTT |
